# Systematic evaluation of clinical efficacy of CYP1B1 gene polymorphism in EGFR mutant non-small cell lung cancer observed by medical image

**DOI:** 10.1515/biol-2022-0688

**Published:** 2023-09-30

**Authors:** Shaofeng Zhang, Danqing Li, Xia Han

**Affiliations:** Xingtai People’s Hospital, Xingtai 054001, Hebei, China

**Keywords:** image observation, CYP1B1 gene polymorphism, EGFR mutation, non-small cell lung cancer, clinical efficacy, medical data

## Abstract

Lung cancer is the cancer with the highest mortality rate and the highest incidence in the world at this stage. Among them, non-small lung cancer is the most common type of lung cancer, and most small cancers have disappeared, which is the optimal time for surgery at the time of diagnosis. To explore and systematically evaluate the clinical efficacy of CYP1B1 gene polymorphism in the treatment of epidermal growth factor receptor (EGFR) Mutant non-small cell lung cancer, this article proposes the principles of lung cancer screening based on CYP1B1 gene polymorphism and polarization imaging and explores the diagnosis and treatment of non-EGFR mutant lung cancer. Based on a large number of medical image data, imageomics can directly reflect the correlation between tumor molecular phenotype and image characteristics by deeply mining some imaging features of the image, which has important value in the early diagnosis of disease, the formulation of personalized treatment plan, and efficacy evaluation and prognosis prediction. A total of 141 NSCLC patients with sensitive EGFR mutation were included in this study, including 101 patients with EGFR single-gene mutation and 40 patients with EGFR multigene mutation coexisting mutation. Both groups of patients were female, aged ≥60 years, no smoking history, no family history of leukemia, adenocarcinoma, lung cancer, stage IV, lymph node metastasis, living, far from metastasis, and ECOG score of 0–2. This study examined the relative number of gene expression and PFS in EGFR multigene co-existing mutations. When the number of mixed genes is 1, 2, and higher, the PFS is 9 months, 8 months, and 6 months, respectively. The PFS time of this group of patients gradually shortened. Therefore, this study examined the benefit of polygenic mutation in estimation by comparing the clinical characteristics of patients with EGFR single-gene mutation and polygenic mutation, to provide measurement of EGFR-TKI and to provide suggestions for future drug selection.

## 1 Introduction

Lung cancer is the malignant tumor with the highest incidence rate and mortality in the world. Non-small cell lung cancer (NSCLC) accounts for 75–80% of primary lung cancer. Due to the lack of a comprehensive early screening and diagnosis system, more than half of NSCLC patients have reached advanced stages when detected, with a 5-year survival rate of less than 30%. In the past 10years, the detection of Epidermal growth factor receptor (EGFR) mutation and the application of Epidermal growth factor receptor Tyrosine kinase inhibitors (EGFR-TKIs) have opened a new era in the treatment of advanced NSCLC. EGFR is a Tyrosine kinase receptor (also known as ErbB-1 or HER1), which is a member of the Epidermal growth factor receptor (HER) family.

The human EGFR gene is located in the short arm 7P12-14 region of Chromosome 7 and consists of 28 Exon. In NSCLC patients, EGFR mutations are mainly concentrated in 18–21 Exon. EGFR gene mutations are mainly dominant in Asian, female, non-smoking, and adenocarcinoma patients. The most common types of mutations are 19 Exon deletion mutations (Del19) and 21 Exon L858R Point mutations, which account for 90% of all EGFR mutation populations. Among advanced NSCLC patients in China, about 50% of them have different types of EGFR gene changes. Many large clinical studies have shown that in advanced NSCLC with EGFR mutant, especially NSCLC patients with 19 Exon deletion or 21 Exon L858R mutant, the progression-free survival (PFS) and overall survival (OS) of the first-generation EGFR-TKIs as the first-line treatment plan are significantly longer than those of the traditional first-line chemotherapy plan based on platinum (carboplatin, cisplatin, Nedaplatin, etc.). However, almost all patients with EGFR-sensitive Mutant advanced NSCLC inevitably develop secondary drug resistance after starting EGFR-TKIs Targeted therapy for 8–24 months, and then disease progression occurs. However, there are also some patients who, despite the presence of EGFR-sensitive mutations, still experience disease progression within the short term (usually within 3 months) of starting treatment with EGFR-TKIs, indicating the presence of primary EGFR-TKIs resistance. At present, the mechanism of acquired drug resistance in first-generation EGFR-TKIs has not been fully elucidated. Current research shows that approximately 50% of the secondary resistance of first-generation EGFR-TKIs is caused by EGFR Exon 20 T790M secondary mutations. Other types of secondary drug resistance mutations include L747S, D761Y, and T854A. Possible mechanisms lead to primary resistance in first-generation EGFR-TKIs include PIK3CA mutations, MET amplification, BIM polymorphism, changes in the PIK3CA-AKT-mTOR signaling pathway, and the transformation from NSCLC to small cell lung cancer.

The meaning of imageomics is to extract high-throughput features from a large number of medical images and carry out feature mining, use statistics and machine learning methods to screen the most valuable imageomics features, and correlate them with other clinical data. It is widely used in a variety of tumor lesions. The research scope includes the genetic phenotype of tumor lesions, tumor grading and staging, efficacy evaluation, and prognosis prediction. The imaging texture features can not only reflect the internal heterogeneity of the tumor but also have some correlation with the biological characteristics of the tumor such as hypoxia, glucose metabolism, and angiogenesis. At present, there are many studies on tumor lesions such as lung, colon cancer, and prostate cancer in imageomics, while the research of head and neck tumors is mainly used to evaluate the prognosis and the changes in biological characteristics and functional information of the lesions after radiotherapy and chemotherapy.

**Figure 1:**
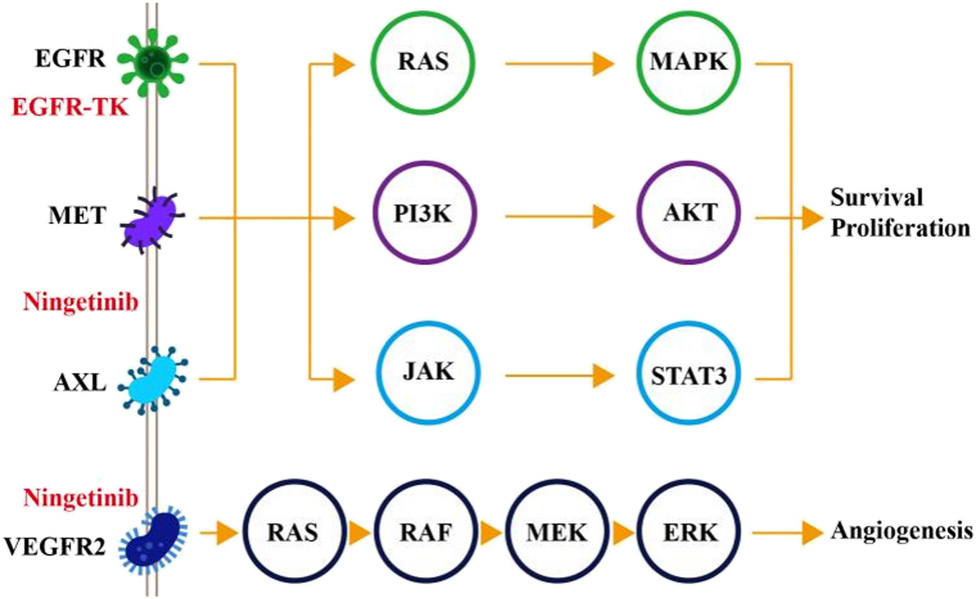
Gene polymorphisms in EGFR mutations in non-small cell lung cancer.

In recent years, cancer has gradually become the disease with the highest mortality rate worldwide. Meanwhile, according to the latest cancer data report released in 2021, there are about 790,000 cases of lung cancer in China every year, and about 631,000 of them die. According to NCCD’s in-depth research on pathogenesis, the oncogenic activity of various oncogenes, including oncogenes, has been linked to the treatment of lung cancer may be difficult due to genetic polymorphisms. In addition, polymorphism of the CYP1B1 gene can be used to study pulmonary disease. Therefore, this article focuses on the study and diagnosis of EGFR, as shown in Figure 1, cell screening, and polarization imaging based on the CYP1B1 gene polymorphism.

## 2 Literature review

In recent years, the TP53 gene has been a hot topic of widespread concern among scholars. At present, different classification standards of TP53 gene mutation have been derived according to the mutation site, mutation nature, and biological effects caused by the mutation. For example, according to the type of mutation, TP53 gene mutations can be divided into Missense mutation and non-Missense mutation; Missense mutation refers to the change of amino acid type and sequence of P53 protein polypeptide chain after base substitution of the codon encoding some amino acid at the mutation site. Except for Missense mutation, all TP53 mutations that can cause changes in the amino acid sequence of P53 protein are collectively referred to as non-Missense mutation (Nonsense mutation, deletions, in frame insertions, cuts, frameshift mutations, etc.). As an emerging field, imageomics is expected to more and more influence the clinical practice of various tumor treatments, optimize the end-to-end diagnosis, treatment, and follow-up chain, and may completely change the traditional classical methods based on macroscopic and microscopic analysis of tumor characteristics. Although small cell carcinoma is usually treated with surgery, 90% of patients will delay surgery once diagnosed, and only about 20% can be cured by surgery. Nearly half of the patients developed local or distant metastases shortly after surgery. After platinum-based chemotherapy is not completed, the survival time is only 5–7 months, and the level of liver cancer treatment is stagnant [1].

Latreille and others designed a mobile phone polarized light microscope and used mobile phone imaging to observe the lesion position of malaria cells in tissues [2]. Iizuka and others took polarized images of animal oocytes at different stages of mitosis and polarized fluorescence imaging of brain tissue by polarizing microscope LCPolScope and analyzed and studied them [3]. Nishio and others used the imaging system to measure the sections of rat liver tissue, obtained the Stokes vector parameters of healthy part and hematoma part, and obtained the Mueller matrix through this vector, so as to quantitatively analyze the polarization characteristic data of rat liver tissue sections [4]. Dammeijer and others used Mueller to analyze and study the anisotropic structure of skeletal muscle [5]. Wakuda and others established different model structures, simulated these structures, obtained Mueller matrix parameters under these models, analyzed them in detail, and applied the analysis results to the study of various heterosexual structures such as skeletal muscle [6]. Wang and others used the polarization method to analyze the cancerous colon, thus proving that the polarization characteristics of cancerous tissue will change compared with normal cancerous tissue [7]. Xu and others studied the polarization characteristics and also quantitatively analyzed it by using gray-level co-occurrence matrix [8]. Lu and others found that the element asymmetry of the birefringent object in the Mueller matrix measured by double-layer sample is higher than that of single-layer sample by using the polarized light imaging method [9].

We have a further understanding of the mechanisms of abnormal proliferation, infiltration, and metastasis of malignant tumor cells at the molecular and cellular levels. The emergence of tumor molecular lead to drugs has brought new hope to patients with advanced lung cancer. Different from traditional cytotoxic drugs, molecular targeted therapeutic drugs take cell receptors, genes, regulatory molecules, and other signal transduction as the boot point, specifically act on these specific sites of tumor cells, and highly selectively kill tumor cells. It is a new drug with high toxicity and side effects and has a very good prospect in the field of tumor treatment [10].

## 3 NSCLC formation process and a representation of polarized light

### 3.1 Review of TCM and argon-helium knife cryotherapy for NSCLC

Although image observation has been applied to many diseases, lung cancer is by far the most widely studied and characteristic malignant tumor through image observation. The research of image observation on lung cancer mainly includes the following aspects: First, it is used for cancer detection, contour and feature description. Because some lung nodules may be small and similar to other structures in the lung (such as blood vessels) or benign processes (such as focal organizing pneumonia), CT-based lung cancer screening has a high false positive rate. Because malignant lung lesions have different image structure features from benign lesions on CT, the image features based on CT have high accuracy in differentiating benign and malignant lesions. Surgery, radiotherapy, and chemotherapy are still the three pillars in the treatment of NSCLC. Molecular targeted therapy and traditional Chinese medicine are also important means in the treatment of NSCLC. About 80–90% of patients with lung cancer can be clearly diagnosed by chest X-ray, CT, bronchoscopy, percutaneous needle aspiration biopsy, positron emission tomography (PET/CT) and other clinical examination methods, but most patients have no obvious symptoms in the early stage, 70–90% have lymph node metastasis and hematostatic spread when they visit the doctor. Even in the lung, brain, bone, liver, adrenal, subcutaneous and other distant metastasis, in the middle and late stage, lost the opportunity of surgery, also missed the best treatment opportunity, can only choose radiotherapy and chemotherapy, Traditional Chinese medicine or targeted therapy. The emergence of new technologies and new treatment ideas provides treatment opportunities for patients in the middle and late stages, which solves many clinical problems to a certain extent and even challenges traditional treatment methods. The status of surgery, radiotherapy and chemotherapy in the comprehensive treatment of NSCLC has been severely challenged, especially for patients with advanced lung cancer, the status of radiotherapy and chemotherapy has begun to shake. Minimally invasive techniques such as argon-helium knife cryoablation and radiofrequency ablation have been able to provide some benefits for patients with advanced lung cancer, offering the opportunity for local “excision,” even in some early patients, as good as surgical excision. Biotargeted therapies such as irressa and tarceva have an absolute advantage in the efficacy of certain NSCLC patients and have replaced radiotherapy and chemotherapy as first-line treatments. However, the effectiveness of modern medical treatments is still limited, with more than 80% of all lung cancer patients dying within a year. According to the 2012 China Cancer Registration Annual Report, lung cancer still occupies the first place in the incidence of malignant tumors in China, with the incidence reaching 53.57/100,000 and the mortality reaching 45.57/100,000. Lung cancer still occupies the first place in the mortality rate of malignant tumors, and the incidence of smoking males over 45 years old is the highest. The ratio of annual deaths to new cases is as high as 0.86, indicating that the cure rate is extremely low. It is still increasing year by year due to food hygiene, smoking, automobile and industrial development of air pollution and other reasons.

### 3.2 Understanding of lung cancer in traditional literature

There is no specific cancer to record lung cancer in traditional Chinese medicine literature, but the earliest record of tumor can be traced back to the Shang Dynasty, and the word “tumor” was recorded on the oracle bones in Yin Dynasty dynasty. “Spirit pivot low stab section really evil” also expounds the “tumor”, think the etiology and pathogenesis of “tumor” is “there has been a knot, qi’s, leave of the body fluid, pathogens, in the condensation, with very easily, even to live forever tumor, by hand press the”, which describes the tumor palpate the texture “nut”, observe its onset is “” day in, and sums up the etiology is not pure. Jin low ge hong’s “elbow backup nasty party” documented malignant tumor of the typical clinical manifestations: “governance died suddenly and violently, belly with objects such as stone, pain like a thorn, crow cried day and night, not conquer one hundred death,” and describes in detail the occurrence, development and prognosis, and believe that “every disease of the firm, to grow more, if you have died, and prison from refractory also, womb disease have a knot, and diet, turn win thin”. Especially the scholar-officials also think its “more” to emerging, conforms to the modern understanding of malignant tumor is a chronic disease, tumor and think it’s difficult to be found early, once found that most tumor has been heavier load and difficult to treat, and against the pain caused by cancer patients, tumor appear to the late development state of cachexia has also done a detailed observation records. The author made a preliminary summary of the clinical transmission and treatment of malignant tumors in General Record of Shengji. Gall Gate in Song Dynasty: “Tumor is just, stay and do not go.

### 3.3 TCM etiology and pathogenesis of lung cancer

The tumor phenotype should be associated with the tumor genotype. Image observation involves qualitative and quantitative phenotypes to identify specific genetic map characteristics. With the introduction of genome map in clinical practice, cancer treatment decisions are increasingly based not only on the clinical characteristics of patients and tumor morphology, but also on individual mutation map. Due to the genetic complexity and heterogeneity of lung cancer, it is suggested that NSCLC with some carcinogenic driving factors can become the target of other indications or clinically developed drugs, which has potential clinical therapeutic significance. There is no direct description of the etiology and pathogenesis of lung cancer in the ancient literature, but from the records of various lung diseases, we can see the ancient philosophers’ elaboration on the etiology, pathogenesis and treatment of lung cancer. “Plain ask – Stabbing method theory” in the “positive gas storage, evil cannot dry, avoid its poison”, how can evil poison play a role in hurting the human body? If the individual vital qi is strong, it will naturally resist evil poison, and when the body vital qi is weak, then “four eight winds to the guest in the meridian, for the six diseases” (“Lingshu – Nine needle treatise”). At the same time, neijing also points out that “internal injury is caused by sorrow and anger, which accumulates into Yi”, and holds that abnormal emotion can also cause internal injury, which leads to lack of positive qi, which is also one of the causes of accumulation. As early as in China’s Qing Dynasty, there are doctors on smoking carcinogenesis explanation, Qing Gu Songyuan thought that “smoke is hot chief, very can hurt Yin, and six sex and seven emotions can fire, phlegm and stasis days long can heat, heat yu days long, into heat poison, heat poison fetor lung, blocking airway, lung qi gongyu, brew cancer poison.” At the same time, his understanding of lung cancer is also focused on heat, heat, that the pathogenesis of the six sexual and seven emotions of heat into a long day of heat poison – heat poison to prevent lung a brew of cancer. This is, to some extent, consistent with modern medicine. Ancient books “miscellaneous diseases source stream Xi Candle” recorded “evil accumulation in the chest, blocked airway, gas can not pass, for. Phlegm. For blood, evil is muscle, evil, both are not made, hence forming shape and had a”, can generally reflect the etiology and pathogenesis of lung cancer: when there is pay for good and evil, evil poison knot in pathogens and vital qi is deficient cannot resist evil, cause lung qi activity, xuan drop loss, TanNing and qi stagnation, collaterals and blood stasis resistance, gradually forming block, eventually sent for lung. For the treatment of lung cancer, Li Zhongzi, a doctor in Ming Dynasty, also gave quite clear guidance in his Medical School Must-Read: “In the beginning, diseases and evil spirits are just beginning to rise, while the positive spirit is still strong and the evil spirit is still shallow. In, the disease gradually, the evil spirit is deeper, the positive spirit is weaker, any and attack and fill; At the end, the disease lasts for a long time, the evil spirit infers, the positive qi dissipates, and then the patient is compensated. “It can be said that up to now, this is still the treatment strategy adopted by most TCM oncologists and even western doctors.

The interaction between host and environmental factors can cause chromosome deletion and inactivation of tumor suppressor genes, and this research is currently in the preliminary stage of research. Common types of lung cancer include adenocarcinoma, squamous cell carcinoma, large cell undifferentiated carcinoma, and small cell lung cancer. Currently, adenocarcinoma is the most common histopathological type, accounting for 30–40% of lung cancer; Squamous cell carcinoma was the most common in the past, but has now been replaced by adenocarcinoma. Large cell undifferentiated carcinoma belongs to the type of large cell tissue, but it is difficult to classify histologically as squamous cell carcinoma or adenocarcinoma. Small cell lung cancer originates from the neuroendocrine cell line, with a faster growth rate, strong invasiveness, and poor prognosis. In the current diagnosis and treatment strategies for lung cancer, it is not important to distinguish between squamous cell carcinoma, adenocarcinoma, or large cell carcinoma. The important difference lies in whether it is small cell lung cancer or NSCLC. As lung cancer tissue continues to grow, and due to differences in tumor growth location and biological characteristics, various clinical symptoms and outcomes can occur:

1. Space occupying effect. The tumor tissue may cause damage to the body through compression of normal tissue structure, such as Venae cavae syndrome or Hoarse voice caused by compression of Recurrent laryngeal nerve.
2. Invading normal tissues directly leads to tissue damage. For example, the erosion of bones and blood vessels by tumors can cause pain and bleeding; Invasion of pleura and pericardium can cause Pleural effusion or pericardial effusion respectively.
3. Blocking the airway can cause obstructive pneumonia, cough, and respiratory distress.
4. The production of bioactive substances leads to paraneoplastic syndrome, such as Vasopressin secretion disorder syndrome (SIADH), hypertrophic pulmonary osteoarthropathy, and hypercalcemia. Lung cancer can also cause consumptive changes in the body, leading to weight loss and fatigue, and its pathogenesis is still unclear.
5. Extrapulmonary metastasis, involving distant organs. The liver, adrenal gland, brain, and bone are common sites of extrapulmonary metastasis in lung cancer.

### 3.4 Representation of polarized light

Polarized light, an optical term. Light is an electromagnetic wave, and electromagnetic waves are transverse waves. The plane formed by the direction of vibration and the direction of light wave progression is called the vibration surface, and the vibration surface of light is limited to a fixed direction, called plane polarized light or line polarized light.

The polarization phenomenon of light can be detected using experimental devices, with P1 and P2 being two identical polarizers. Observing natural light (such as light or sunlight) directly through a polarizer p1, although the light passing through the polarizer becomes polarized light, it cannot be detected by the human eye due to its inability to distinguish polarized light. If we fix the orientation of polarizer P1 and slowly rotate polarizer P2, we can observe periodic changes in the intensity of transmitted light as P2 rotates, and the luminous intensity will repeatedly decrease from maximum to darkest every 90° rotation; Continuing to rotate P2 will gradually increase the light intensity from close to zero to its maximum. From this, it can be seen that the transmitted light through P1 has different properties from the original incident light, indicating that the vibration of the transmitted light through P1 does not have symmetry in the direction of propagation. After passing through a polarizer, natural light changes into light with a certain vibration direction. This is because there is a characteristic direction in the polarizer, called polarization direction. The polarizer only allows vibrations parallel to the polarization direction to pass through, while filtering out light that vibrates perpendicular to that direction. Through the transmission of light through a polarizer, its vibration is limited to a certain direction of vibration. We call the first polarizer P1 the “polarizer”. Its function is to convert natural light into polarized light, but the human eye cannot distinguish polarized light. It is necessary to rely on the second polarizer P2 for inspection. Rotate P2, when its polarization direction is parallel to the polarization plane of polarized light, the polarized light can pass smoothly, and there is brighter light behind P2. When the polarization direction of P2 is perpendicular to the polarization plane of polarized light, polarized light cannot pass through and darkens behind P2. The second polarizer helps us identify polarized light, hence it is also known as a “polarizer”.

#### 3.4.1 Jones vector

Typically, polarized light is elliptically polarized light. If the light is presented in the z-axis direction, the projection of a beam of elliptically polarized light on the coordinate axis of the XY plane is shown in formulas (1) and (2).

(1) \[{E}_{x}={E}_{0x}{e}^{-i(\omega t-kz+{\phi }_{0x})}={E}_{0x}{e}^{-i\omega t}{e}^{i{\phi }_{0x}},]\]

(2) \[{E}_{y}={E}_{0y}{e}^{-i(\omega t-kz+{\phi }_{0y})}={E}_{0y}{e}^{-i\omega t}{e}^{i{\phi }_{0y}}.]\]

The common factor is removed, and the projection expression is expressed by complex number and amplitude, which is shown in formulas (3) and (4).

(3) \[{E}_{x}={E}_{0x}{e}^{i{\phi }_{0x}},]\]

(4) \[{E}_{y}={E}_{0y}{e}^{i{\phi }_{0y}}.]\]

American physicists have found that the above formula can be expressed more efficiently by introducing a column matrix composed of mutually orthogonal components. This expression is called Jones vector. Its expression is shown in formula (5).

(5) \[\left[\begin{array}{c}{E}_{x}\\ {E}_{y}\end{array}\right]=\left[\begin{array}{c}{E}_{0x}{e}^{i{\phi }_{0x}}\\ {E}_{0y}{e}^{i{\phi }_{0y}}\end{array}\right].]\]

Therefore, the intensity of polarized light can be expressed by formula 6.

(6) \[I={| {E}_{x}| }^{2}+{| {E}_{y}| }^{2}={E}_{0x}^{2}+{E}_{0y}^{2}.]\]

#### 3.4.2 Stokes vector

The Stokes vector method uses four metrics to determine the light intensity in different directions and states, and the resulting data is shown in equation (7).

(7) \[S=\left[\begin{array}{c}I\\ Q\\ M\\ V\end{array}\right]=\left[\begin{array}{c}{F}_{x}^{2}+{F}_{y}^{2}\\ {E}_{x}^{2}-{E}_{y}^{2}\\ 2{E}_{n}{E}_{x}\hspace{.25em}\cos \hspace{.25em}\sigma \\ 2{E}_{x}{E}_{y}\hspace{.25em}\sin \hspace{.25em}\sigma \end{array}\right]=\left[\begin{array}{c}{N}_{x}+{N}_{y}\\ {N}_{x}-{N}_{y}\\ {Y}_{+{45}^{\circ }}-{Y}_{-{45}^{\circ }}\\ {B}_{R}-{B}_{L}\end{array}\right].]\]

Table 1 provides the Stokes vectors for different graphs with different polarization states.

**Table 1:** Several special Stokes vector parameters

Special Stokes vector	*M*	*Q*	*U*	*N*
Level	2	1	1	0
Vertical line	2	6	0	0
45° plate polarization	3	0	2	1
Right circular polarization	8	0	4	2

According to Stokes not, some polarizations are not available, such the expression for the degree of polarization is shown in equation (8).

(8) \[P=\frac{\sqrt{{Q}^{2}+{U}_{2}+{I}^{2}}}{I}.]\]

#### 3.4.3 Mueller matrix

Since the Stokes vector can be a feature, the incident problem and the light emitted after entering the polarizer can be represented by the Stokes vector. The relationship between light events, light output, and past problems is shown in formula (9)

(9) \[s=ms.]\]

Formula (9) is expressed in the form of matrix, and the result is shown in formula (10).

(10) \[\left[\begin{array}{c}{S}_{1}^{^{\prime} }\\ {S}_{2}^{^{\prime} }\\ {S}_{3}^{^{\prime} }\\ {S}_{4}^{^{\prime} }\end{array}\right]=\left[\begin{array}{c}\begin{array}{cccc}\text{N} {y}_{12} {n}_{13} {n}_{14}\end{array}\\ \begin{array}{cccc}n {n}_{22} {n}_{23} {n}_{24}\end{array}\\ \begin{array}{cccc}{n}_{31} {n}_{32} {n}_{33} {n}_{34}\end{array}\\ \begin{array}{cccc}{m}_{n41} {n}_{42} {n}_{43} {n}_{44}\end{array}\end{array}\right]\left[\begin{array}{c}{S}_{1}\\ {S}_{2}\\ {\text{Y}}_{3}\\ {\text{Y}}_{4}\end{array}\right].]\]

Nueller matrix is shown in formula (11).

(11) \[M=\left[\begin{array}{c}\begin{array}{cccc}{m}_{11} {n}_{12} {n}_{13} {n}_{14}\end{array}\\ \begin{array}{cccc}{m}_{21} {n}_{22} {n}_{23} {n}_{24}\end{array}\\ \begin{array}{cccc}{m}_{31} {n}_{32} {n}_{33} {n}_{34}\end{array}\\ \begin{array}{cccc}{m}_{41} {n}_{42} {n}_{43} {n}_{44}\end{array}\end{array}\right].]\]

There are many parameters to describe the polarization characteristics by Mueller matrix, among which the degree of polarization and polarization difference are the most commonly used. The expression of degree of polarization is shown in formula (12).

(12) \[P=\frac{{I}_{p}}{{I}_{\text{t}}}=\frac{{I}_{\max }-{I}_{\min }}{{I}_{\max }+{I}_{\min }}.]\]

Degree of polarization (DOP) parameters and polarization difference (DP) parameters are an important standard to characterize the polarization characteristics of samples [11]. These parameters were first used in polarization imaging methods and applied to clinical diagnosis [12]. Because the optical imaging method has some limitations in detecting tissue, and the polarization imaging method can improve the imaging contrast of biological tissue, it is often used with skin tissue detection. Degree of polarization and polarization difference can characterize the polarization characteristics of substances [13]. The expression of degree of polarization obtained by Nueller matrix is shown in formula (13).

(13) \[DOP=\frac{{m}_{21}+{m}_{22}}{{m}_{11}+{m}_{12}}.]\]

The Mueller matrix expression of polarization difference is shown in formula (14).

(14) \[DP={m}_{21}+{m}_{22}.]\]

#### 3.4.4 Gray associative matrix

**Figure 2:**
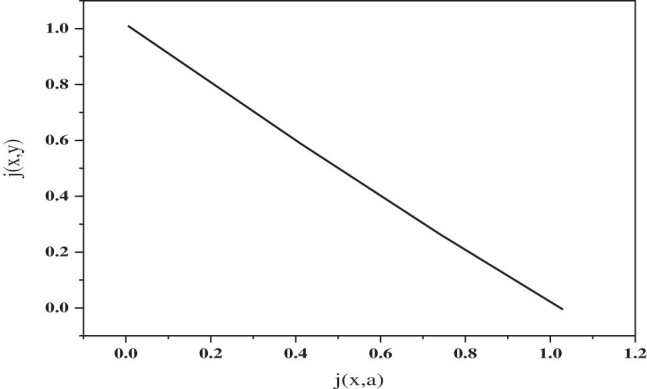
Principle of gray level co-occurrence matrix.

So it is generally effective to use pattern texture measurement. In general, the texture formed recursively in the spatial position of the gray value distribution in the graph is determined as the texture, that is, the texture, and the texture has intertwined features, that is, there may be some gray interactions. between some two pixels [14]. The grayscale phase co-occurrence matrix is a statistical matrix that calculates the occurrence time of pairs of grayscale values that have the same grayscale value in a certain direction and another point at a distance [15]. The principle is shown in Figure 2.

## 4 Experiment

Image observation can quantitatively analyze and fully mine the relationship between pixels and spatial distribution in the image, supplement the traditional morphological imaging features, and can be used as a useful imaging diagnostic tool for early prediction of EGFR gene mutations in patients with NSCLC. The heterogeneity of lung cancer is related to many factors, such as changes in growth factors and angiogenesis factors, mutations in related gene subtypes or changes in tumor microenvironment. The tumor morphology, internal density and other characteristics provided by the image can indirectly provide relevant information from the overall level for tumor heterogeneity analysis. The CT-based imageomics can help to improve the sensitivity and accuracy of differentiating benign and malignant lung lesions, non-invasive identify lung cancer of different pathological types, quantitatively predict the mutation status of lung cancer gene, and evaluate the heterogeneity of lung cancer to help clinical personalized treatment Predict and evaluate treatment response and prognosis of lung cancer patients. 141 patients were finally selected for inclusion and exclusion according to the criteria. Important data on these patients included gender, age, important site, disease type, treatment stage, lymph node metastasis, distant metastasis, smoking history, tumor, and family history of tumor (according to ECoG scoring criteria). EGFR mutant strains, coexisting genes, EGFR-TKI therapy, and post-treatment vaccines were documented and regularly followed [16].

Tissue or body fluids (blood, urine, pleural effusion, etc.) have EGFR altered by NGS detection Sensitivity, with or without other gene mutations; Receiving EGFR-TKI; Viruses can be detected according to RECIST version 1.1 and regularly checked in our hospital; Adults (age ≥18 years old) are generally in good condition [17].

Exclusion criteria: 1. Have serious organic diseases such as liver and kidney function and heart failure; 2. Complicated with other malignant tumors or serious infection; 3. Those who have taken EGFR-targeted drugs before advanced stage; 4. Failure to review regularly, incomplete treatment and review information, or loss of follow-up; 5. Taking targeted drugs combined with other treatment schemes that can significantly affect the prognosis; 6. Irregular medication or self-discontinuation of targeted drugs [18].

## 5 Clinical characteristics of patients with EGFR single gene mutation and multi gene coexisting mutation

### 5.1 Clinical features

The clinical characteristics of the two groups are as follows (Table 2). In the same point, both groups were female (single mutation group vs coexisting mutation group, 69.3% vs 67.5%); Age ≥60 years old (single mutation group vs coexisting mutation group, 63.4% vs 60.0%); No smoking history (single mutation group vs coexisting mutation group, 74.3% vs 82.5%); No family history of tumor (single mutation group vs coexisting mutation group, 85.1% vs 72.5%); Adenocarcinoma (single mutation group vs coexisting mutation group, 98% vs 97.5%); right lung (single mutation group vs coexisting mutation group, 61.4% vs 57.5%); Stage IV (single mutation group vs coexisting mutation group, 68.3% vs 70.0%); Lymph node metastasis (single mutation group vs coexisting mutation group, 57.4% vs 67.5%); Single site distant metastasis (single mutation group vs coexisting mutation group, 53.5% vs 40.0%); ECoG 0–2 points (single mutation group vs coexisting mutation group, 90.1% vs 87.5%) [19]; First generation EGFR-TKIs (including ektinib, gefitinib, and erlotinib) treatment (single mutation group vs coexisting mutation group, 96.1% vs 87.5%); No T790M mutation was common after treatment (single mutation group vs coexisting mutation group, 88.1% vs 85.0%) [20]. There were more exon 19 deletions in the EGFR single gene mutation group (single mutation group vs coexisting mutation group, 55.4% vs 40.0%); The EGFR polygenic mutation group had more mutations in exon 21 L858R (single mutation group vs coexisting mutation group, 40.6% vs 52.5%). As shown in Table 2.

**Table 2:** Clinical characteristics of EGFR single gene mutation group and EGFR multigene coexistence mutation group

EGFR multigene coexistence mutation		EGFR single-gene mutation	*X* ^2^	*p*
Gender	Male 31	13	0.016	0.29
	Female 70	27		
Age	>60	18	0.2369	3.69
	<70	28		
Smoking history	Yes	26	0.269	4.21
	No	30		
clinical stages	32	12	1.236	8.36
	69	15		

### 5.2 Gene distribution of EGFR multigene coexisting mutation group

**Figure 3:**
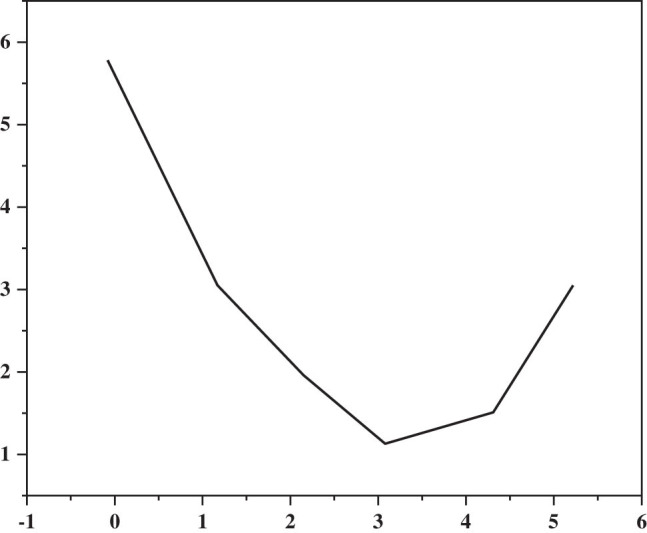
Distribution of coexisting gene mutations in patients with EGFR mutation-positive advanced NSCLC.

The gene distribution of 40 advanced NSCLC patients with EGFR multigene co-mutation is as follows (3). Exon 19 was removed in 16 cases, and exon 21 L858R was replaced in 21 cases. Other changes included 2 cases of exon 21 l861q and 1 case of exon 18 g719a. Coexisting gene mutations were higher at the exon 21 L858R transition (exon 19 versus exon 21, 40% versus 52.5%) [21]. Among all gene mutations, TP53 mutation is the highest. Of these, 20 patients switched to TP53, accounting for 50% of the change in this group, followed by BIM (10%) and PIK3CA (10%) mutations. In the gene transfer data of this study, 20 patients were associated with only 1 mutation, including 7 with TP53 mutation, 3 with BIM removal, 2 with ros1 alteration, and 2 with ALK altered 3 times. Amplification, 1 case of PIK3CA mutation and 1 case of HER2 amplification mutation. Another 20 patients were diagnosed with multiple genetic mutations, as shown in Figure 3.

### 5.3 Relationship between EGFR coexisting gene mutations and the efficacy of EGFR-TKIs

The performance measures for the two patient groups in this study are shown in Table 3: CR was not achieved in either group. The ORR of EGFR gene mutation group and EGFR multigene co-mutation group were 31.7 and 45%, respectively.

**Table 3:** Comparison of curative effects

Efficacy evaluation	EGFR single gene mutation	EGFR polygenic coexistence mutation	*P*
PD	6 (6.0%)	3 (6.0%)	2.000
PR	31 (32.3%)	21 (34.0%)	0.213
S D	54 (34.7%)	23 (45.0%)	0.258
ORR	21.9%	55%	0.257

### 5.4 Relationship between EGFR coexisting gene mutations and PFS after EGFR-TKIs treatment

**Figure 4:**
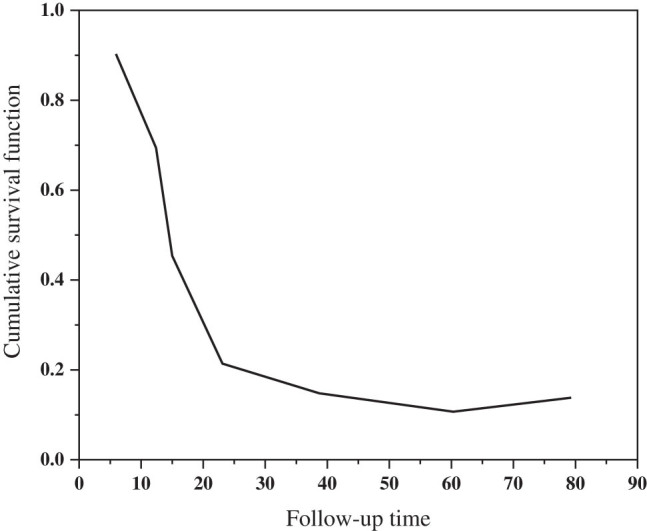
PFS curve of EGFR single gene mutation group and EGFR multigene coexistence mutation group.

Changes in EGFR polygenic mutations after EGFR group-mutated PFS and EGFR-TKI treatment are shown in Figure 4. The mean PFS for EGFR mutations was 12 months, and the mean PFS for the EGFR polygenic co-mutation group was 7 months. Univariate screening determined the patient’s age, sex, smoking history, tumor family history, disease, ECoG score, and type of EGFR change (only exon 19 was removed and L858R was identified). The results are shown in Table 4. Family history of leukemia and treatment level were found to be negative for PFS (*P*-values 0.019 and 0.014, respectively), with no differences in PFS with respect to age, sex, smoking history, lymph node metastasis, and ECoG score. Many tests include family history of the EGFR group, stage of treatment, and cancer. The results showed that the association of EGFR, that is, the combination of multiple gene mutations, was an independent effect on negative PFS (*P* = 1.017, HR = 2.705, 96%). CI: 1.101–3.641), see Table 5.

**Table 4:** Univariate analysis of PFS after EGFR-TKIs treatment

Factor		Frequency	*X* ^2^	*P*
Gender	Male	47	0.043	0.853
	Female	89		
Age	<60	61	0.003	0.965
	≥60	92		
Smoking history	Yes	34	0.021	0.972
	No	104		
Family history of tumor	Yes	24	5.098	0.021
	No	109		

**Table 5:** Relationship between multiple factors and PFS by Cox regression analysis

						95% CI	
	*β*	Standard deviation	Waldx^2^	*P*	HR	Lower limit	Upper limit
EGFR grouping	0.456	0.213	5.674	0.019	1.431	1.232	2.478
Family history of tumor	−0.386	0.295	2.871	0.142	0.570	0.246	1.109
Stages	−0.32	0.254	1.68	0.198	0.612	0.655	1.466

### 5.5 Relationship between EGFR coexisting gene mutation and OS after EGFR-TKIs treatment

**Figure 5:**
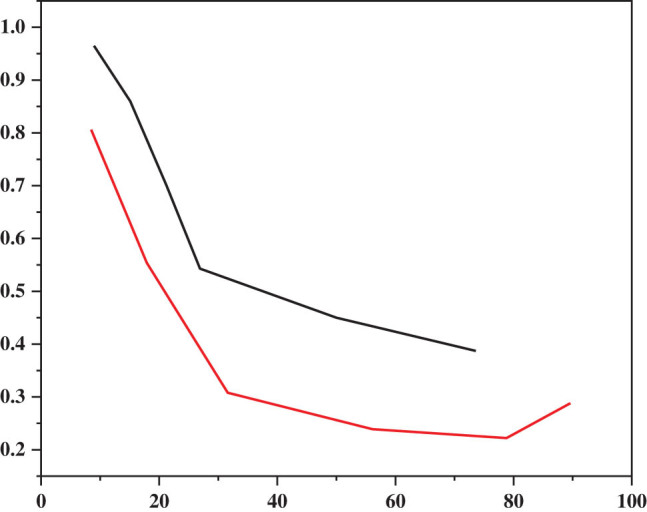
OS curve of EGFR single gene mutation group and EGFR multigene coexistence mutation group.

The OS after EGFR-TKI treatment in the EGFR single-gene mutation group and the EGFR multigene co-mutation group is shown in Figure 5. 26 mm vs 26 mm 26 mm 21 months), but there is no difference between the two groups (*P* = 0.284) in outcomes including patient age, sex, smoking history, family history, health status, ECOG score, and type of EGFR change (only two exon 19 variants and L858R were identified), as shown in Table 6. Treatment levels were found to be negative for OS (*P* = 0.017), age, sex, smoking history, family history of tumors, ECOG score, and EGFR changes. EGFR groups and treatment stages are included in various tests. EGFR group was not associated with OS (*P* = 1.04, HR = 1.615, and 96% CI: 1.122–2.552).

**Table 6:** Univariate analysis of OS after EGFR-TKI treatment

Factor		Frequency	*X* ^2^	*p*
Gender	Male	46	0.767	0.342
	Female	89		
Age	<60	45	0.156	0.768
	≥60	91		
Clinical stages	Phase III	45	5.378	0.019
	Phase IV	98		
Lymph node metastasis	Yes	89	0.131	0.782
	No	57		

### 5.6 Relationship between the number of genes and PFS

**Figure 6:**
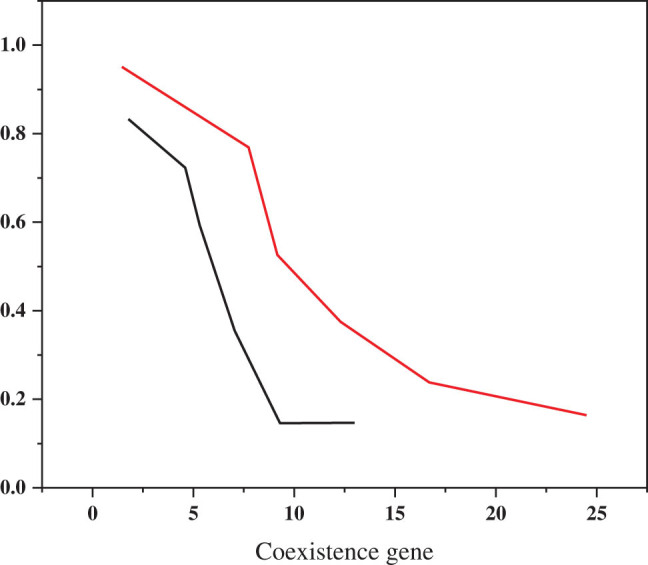
Relationship between the number of coexisting gene mutations and PFS.

This study examined the relative numbers of gene expression and PFS in a panel of EGFR polygenic coexisting mutations. The results are shown in Figure 6. When the number of mixed genes was 1, 2, and higher, the PFS was 9, 8, and 6 months, respectively. The PFS time was gradually shortened in this group.

## 6 Discussion

Combining molecular biological characteristics with imaging characteristics is also the development trend of tumor diagnosis and treatment in the future. In recent years, the efficacy of chemotherapy has reached a bottleneck. The median OS is about 7–12 months, and the 2-year survival rate is only 10–11%.genomics and molecular detection technology, the control targets of gene processing have gradually increased, especially the discovery of EGFR-producing genes. EGFR is widely distributed, in fibroblasts and other cells, and plays an important role in cell proliferation and differentiation. The rate of change in NSCLC patients is approximately 19–50%. The most common variants are exon 30 deletions and exon 41L858R converters. Because EGFR-TKIs are unique and rare, and their therapeutic effects are superior to therapeutic drugs, they have become the first-line treatment for patients with EGFR-sensitive and sensitive NSCLC. Earlier studies thought the genes were cohesive, but in recent years, patients with multiple mutations have been identified. However, multigene co-mutation in NSCLC patients is very low, accounting for only 1–6%. Currently, implementation of the T790M variant EGFR-TKI-related expansion has been declared inappropriate; 18 patients were diagnosed with the T790M mutation and developed immunity after developing. However, the effects and correlations of polygenic mutations on the efficacy of EGFR-TKIs are still unclear, and some studies have been conducted. Of the 141 patients, 93.6% received first-generation EGFR-TKI therapy. Therefore, this study examined the benefit of polygenic mutation in estimation by comparing the clinical characteristics of patients with EGFR single-gene mutation and polygenic mutation. To provide measuring of EGFR-TKI, to provide suggestions for future drug selection.

## 7 Conclusion

This article presents the principle of lung cancer screening based on CYP1B1 gene polymorphism and polarization imaging and explores the diagnosis and treatment of non-EGFR mutant lung cancer. Based on a large number of medical imaging data, imaging omics directly reflects the correlation between tumor molecular phenotype and imaging features by deeply mining some imaging features and is of great value for early diagnosis, the formulation of personalized treatment plan, efficacy evaluation, and prognosis prediction. The clinical data of this study showed that the incidence of EGFR polygenic coexisting mutations in non-small tumors was about 8.7%, while the incidence of TP53, BIM, PIK3CA, and other factors such as age, gender, smoking history, disease and other factors was about 8.7%. Tumor family history, treatment level, lymph node metastasis, EGFR mutation type, and EGFR mutation immune score combined several EGFR groups, but both groups were mostly female, nonsmoking, ≥60 years old, and leukemia patients. The clinical characteristics of 299 patients with EGFR mutation and 21 patients with EGFR mutation were compared, and the difference was not significant. This study shows the relative amount of gene expression and PFS in coexisting mutations in EGFR. The results are shown in Figure 6. When the number of admixed genes was 1, 2, and higher, the PFS was 9, 8, and 6 months, respectively. The PFS time was gradually shorter in the patients in this group. Considering the reasons for the differences between studies, first, sample size; second, Guibert’s study focused on all lung cancer patients, and it can calculate the time it takes them to go through multiple changes. The study material for this study is based on EGFR-mutant NSCLC patients with or without other genetic mutations. The research potential of the two studies was different, so the results were different. In the future, with the further maturity of image observation, the diversification of research queues, the improvement of large samples and multi-center databases, and the update of machine learning algorithms, the above problems can be effectively solved. How to explore new targets and develop corresponding targeted drugs. At present, only a few molecular targets of lung cancer have been confirmed to be clinically significant, and corresponding targeted inhibitors have been approved for marketing. Another part of the targets and corresponding inhibitors are still in the clinical research stage, but more valuable drug targets have not yet been found. It is believed that in the future, with the promotion and application of high-throughput sequencing and the rapid development of molecular biology technology, as well as the in-depth discussion of clinical research and the invention of the mechanism of the occurrence and development of lung cancer, more unknown or immature targets will be gradually understood, and the corresponding research and development of targeted drugs and clinical applications will also make significant progress, and the Targeted therapy of lung cancer will inevitably move towards precision, individualization, and maturity, Thus, more and more patients will truly benefit from survival. Image observation will also have a broader application prospect in the molecular diagnosis and treatment of lung cancer.
